# 1200. Evaluation of Pharmacist-Driven Azithromycin Dose Optimization Protocol for Respiratory Tract Infections as an Antimicrobial Stewardship Initiative

**DOI:** 10.1093/ofid/ofad500.1040

**Published:** 2023-11-27

**Authors:** Satwinder S Kaur, David T Adams, Casey L Schrank

**Affiliations:** Texas Health Harris Methodist Hospital Fort Worth , Dallas, Texas; Texas Health Harris Methodist Hospital Fort Worth, Fort Worth, Texas; Texas Health Harris Methodist Fort Worth, Burleson, Texas

## Abstract

**Background:**

Azithromycin is a macrolide antibiotic commonly used empirically to treat atypical pathogens in CAP and acute COPD exacerbations. Due to its longer half-life and high tissues concentrations, a total treatment dose of 1.5 g (given over either 3 or 5 days) provides therapeutic exposure lasting approximately 10 days. Multiple randomized studies demonstrated similar treatment efficacy between 3 days and 5 days of azithromycin. Due to the increased rates of macrolide-resistant *Streptococcus pneumoniae* strains, shorter courses of azithromycin may decrease the emergence of more bacterial resistance, improve patient compliance, and decrease antibiotic-associated adverse events.

**Methods:**

A retrospective cohort study conducted from March 2022 to March 2023 at Texas Health Harris Methodist Hospital Fort Worth. Pharmacists automatically adjusted azithromycin orders to a duration of 3 days and a dose of 500 mg for CAP and acute COPD exacerbations. Patients were excluded if treatment indications were for prophylaxis, mycobacterial disease, or Legionnaires disease. The primary outcome was azithromycin duration of therapy (DOT) after protocol implementation and secondary outcomes included 30-day all-cause in-hospital mortality and 30-day hospital readmission.

**Results:**

507 patients were included in the final analysis with 267 patients in the pre- and 240 patients in the post-group. After protocol implementation, a statistically significant reduction in mean azithromycin DOT was observed in the post- compared to the pre-group (3 days vs 5 days), *p* < .0001. No increase in 30-day all-cause in-hospital mortality (2.1% vs. 7.1%, *p* = .008) or 30-day hospital re-admission rates (5.4% vs. 15.3%, *p* = .001) were observed in the post- compared to the pre-group.

**Conclusion:**

Based on the results of this study, implementation of a pharmacist-driven azithromycin dose optimization protocol is an effective antimicrobial stewardship initiative that can decrease unnecessary azithromycin DOT. The 3-day duration of high dose azithromycin was not associated with increased rates of mortality or hospital re-admissions compared to longer durations.

**Disclosures:**

**All Authors**: No reported disclosures

